# Increased Cerebrospinal Fluid Uric Acid Levels in Guillain–Barré Syndrome

**DOI:** 10.3389/fneur.2020.589928

**Published:** 2020-11-12

**Authors:** Sheng-Hui Chang, Xiao-Bing Tian, Jing Wang, Ming-Qi Liu, Chen-Na Huang, Yuan Qi, Lin-Jie Zhang, Chun-Li Gao, Da-Qi Zhang, Li-Sha Sun, Li Yang

**Affiliations:** ^1^Department of Neurology, Tianjin Neurological Institute, Tianjin Medical University General Hospital, Tianjin, China; ^2^Department of Neurology, The First Affiliated Hospital of Hainan Medical University, Haikou, China; ^3^Department of Clinical Laboratory Center, Tianjin Medical University General Hospital, Tianjin, China

**Keywords:** Guillain-Barré syndrome, acute inflammatory demyelinating polyneuropathy, uric acid, cerebrospinal fluid, purine metabolism

## Abstract

Uric acid (UA) is a natural scavenger for peroxynitrite and can reflect antioxidant activity and oxidative stress in several neurological disorders. Changes in serum and cerebrospinal fluid (CSF) levels of UA have been reported in patients with multiple sclerosis and neuromyelitis optica spectrum disorders. The levels of UA in CSF are relatively poorly understood in patients with Guillain–Barré syndrome (GBS). It remains unclear whether UA can play an antioxidant role and reflect oxidative stress in GBS. The purpose of this study is to investigate CSF and serum UA levels in patients with GBS and their relationship with clinical characteristics. The CSF and serum UA levels were detected in 43 patients with GBS, including 14 acute inflammatory demyelinating polyneuropathy (AIDP), 6 acute motor axonal neuropathy (AMAN), 13 with acute motor and sensory axonal neuropathy (AMSAN), 7 Miller Fisher syndrome (MFS), and 3 unclassified, and 25 patients with non-inflammatory neurological disorders (NIND) as controls. Moreover, serum UA levels were also detected in 30 healthy controls. The levels of UA were measured using uricase-based methods with an automatic biochemical analyzer. CSF UA levels were significantly increased in patients with GBS (*p* = 0.011), particularly in patients with AIDP (*p* = 0.004) when compared with NIND. Among patients with GBS, CSF UA levels were higher in those with demyelination (*p* = 0.022), although the difference was not significant after multiple testing correction. CSF UA levels in GBS were positively correlated with serum UA levels (*r* = 0.455, *p* = 0.022) and CSF lactate (*r* = 0.499, *p* = 0.011). However, no significant correlations were found between CSF UA levels and GBS disability scores. There were no significant differences in serum UA levels among GBS, NIND, and healthy controls. These results suggest that CSF UA may be related to the pathogenesis of demyelination in patients with GBS and may be partially determined by serum UA and the impaired blood–nerve barrier.

## Introduction

Uric acid (UA) is the final metabolite of purine degradation in the human. As a natural scavenger for peroxynitrite and oxygen radicals, UA is thought to be one of the most important antioxidants and responsible for nearly two-thirds of the total plasma antioxidant activity (1, 2). The role of UA in the nervous system has attracted widespread attention during the past 30 years (3–5). The neuroprotective effect of UA in the central nervous system (CNS) has been identified by many investigations in animal models of multiple sclerosis (MS), ischemic brain injury, and spinal cord injury (3, 4, 6, 7). By diminishing oxidative damage in brain, UA may play a potential therapeutic role in the CNS diseases (8–11). Several studies have shown inconsistent UA concentrations in neurological diseases. The UA levels in MS and other neurological diseases have been reported either increased or decreased when compared with controls in different studies (8, 12–17). It has been reported that reduced serum UA levels are linked to the development and progression of neurological diseases, including MS, neuromyelitis optica (NMO), Alzheimer's disease, and Parkinson's disease (8, 12–14). On the other hand, increased levels of UA and purine compounds in cerebrospinal fluid (CSF) and serum of MS patients were reported to be associated with increased purine and adenosine triphosphate (ATP) catabolism and energy imbalance (15). A study in neuromyelitis optica spectrum disorders (NMOSDs) discovered increased CSF UA levels during relapse, suggesting oxidative stress and excitotoxicity in these patients (16). Therefore, UA levels may also reflect oxidative stress and ATP metabolism in neurological diseases (17). These inconsistent findings point to a more complex relationship between UA and neurological disorders. On the one hand, it remains unclear whether the reduced concentration of serum UA is a cause or a consequence of antioxidant in neurological diseases (12). On the other hand, further studies are required to discuss whether the elevation of UA levels in neuroinflammatory diseases is related to oxidative stress and ATP degradation (15–17).

Guillain–Barré syndrome (GBS) is an acute onset autoimmune inflammatory peripheral neuropathy. The clinical features of GBS are characterized by rapid progressive symmetrical muscle weakness, decreased tendon reflexes or areflexia, and neuropathic pain (18). Abnormal immune attacks against peripheral nerves and spinal nerve roots are usually associated with precursor infections or other immune stimulation, which often lead to demyelination or axonal damage (19). There are several clinical variants and subtypes in GBS including mainly demyelinating form: acute inflammatory demyelinating polyneuropathy (AIDP); mainly axonal damage form: acute motor axonal neuropathy (AMAN) or acute motor and sensory axonal neuropathy (AMSAN), and Miller Fisher syndrome (MFS) variant, which is characterized by sensory ataxia, oculomotor weakness, and areflexia (20). Antibodies against gangliosides are generally accepted immunopathogenesis in axonal and MFS variants, whereas unknown antigens, complement activation, and macrophage scavenging are considered to be involved in demyelinating form of GBS (20). In addition, oxidative stress and free radical toxicity have been proven to contribute to the pathogenic mechanism of GBS (21–24). However, compared with MS, NMO, and other diseases in CNS, there are relatively less research focusing on the levels of UA in GBS. Patients with GBS showed reduced serum UA levels compared with healthy controls in the study of Peng et al., but the statistical test of serum UA levels between patients with GBS and healthy controls was not performed (25). Su et al. demonstrated reduced serum levels of UA in GBS patients, but without data for CSF, and proposed UA as a protective factor for GBS (26). However, in the study of Becker et al. (27), urate levels of 18 patients with GBS were higher in CSF samples but did not differ significantly in the serum compared with controls. Unfortunately, in their study, patients with GBS lacked clinical information and were not divided into subtypes of GBS probably due to the small sampling size (27). In the present study, we hypothesized that UA levels in CSF may be increased in patients with GBS, reflecting oxidative stress and ATP metabolism. In addition, it was assumed that CSF UA levels might vary among subtypes of GBS. This study investigated CSF and serum UA levels in 43 patients with GBS and its subtypes and estimated the relationship between CSF UA levels and clinical features.

## Materials and Methods

### Patients

This study involved 43 patients with GBS who were hospitalized in the Neurology Department of Tianjin Medical University General Hospital from March 2018 to October 2019. Patients with GBS were diagnosed according to the Brighton criteria (28) by experienced neurologist, and the level of diagnostic certainty is shown in Table 1. According to Ho's criteria (29) and Brighton criteria (28), all the GBS patients were divided into four subtypes based on electrophysiological and clinical characteristics: AIDP (*n* = 14), AMAN or AMSAN (*n* = 19, including 6 with AMAN and 13 with AMSAN), MFS (*n* = 7), and unclassified (*n* = 3). Twenty-five age- and sex-matched patients with non-inflammatory neurological disorders (NINDs) were enrolled as controls, including seven patients with subacute combined degeneration, four patients with mental disorders, four patients with cerebrovascular disease, two patients with cerebral venous sinus thrombosis, two patients with myasthenia gravis, two patients with nutritional and vitamin deficiency neuropathy, two patients with cervical spondylosis, one patient with migraine, and one patient with spinal cavernous hemangioma. Thirty healthy controls from the Health Center of Tianjin Medical University General Hospital were also enrolled in this study. Cases including patients with GBS, NIND controls, and healthy controls who had been treated with acetylsalicylic acid, antitubercular drugs, thiazide diuretics, or other drugs that could affect UA levels (30) within the last 12 weeks, as well as cases with gout, diabetes mellitus, and hepatic or renal disorders were excluded from this study.

**Table 1:** Demographic and clinical features in patients with GBS, NIND, and healthy controls.

	**GBS**	**AIDP**	**AMAN/AMSAN**	**MFS**	**Unclassified**	**NIND**	**HC**
	**(*n* = 43)**	**(*n* = 14)**	**(*n* = 19)**	**(*n* = 7)**	**(*n* = 3)**	**(*n* = 25)**	**(*n* = 30)**
Age (years), mean ± SD	47.35 ± 16.62	52.14 ± 21.04	43.68 ± 13.47	48.57 ± 15.26	45.33 ± 17.04	43.48 ± 17.59	50.53 ± 14.47
Gender (female/male)	22/21	5/9	12/7	3/4	2/1	13/12	14/16
Preceding infections, *n* (%)	24 (55.8%)	7 (50.0%)	12 (63.2%)	3 (42.9%)	2 (66.7%)	–	–
Cranial nerve involvement, *n* (%)	19 (44.2%)	5 (35.7%)	4 (21.1%)	7 (100.0%)	3 (100.0%)	–	–
GBS disability scores, median (IQR)	2.00 (2.00, 3.00)	3.00 (2.00, 3.25)	2.00 (2.00, 4.00)	1.00 (1.00, 2.00)	2.00 (2.00, 3.00)^**#**^	–	–
Anti-ganglioside antibodies, *n* (%)	28 (65.1%)	7 (50.0%)	14 (73.7%)	6 (85.7%)	1 (33.3%)	–	–
Albuminocytologic dissociation, *n* (%)	39 (90.7%)	13 (92.9%)	17 (89.5%)	6 (85.7%)	3 (100.0%)	–	–
**Brighton criteria level**							
Level 1, *n* (%)	35 (81.4%)	13 (82.9%)	17 (89.5%)	5 (71.4%)	0 (0.0%)	–	–
Level 2, *n* (%)	8 (18.6%)	1 (7.1%)	2 (10.5%)	2 (28.6%)	3 (100.0%)	–	–
Level 3, *n* (%)	0 (0.0%)	0 (0.0%)	0 (0.0%)	0 (0.0%)	0 (0.0%)	–	–
Level 4, *n* (%)	0 (0.0%)	0 (0.0%)	0 (0.0%)	0 (0.0%)	0 (0.0%)	–	–

# *Data presented as median (range) instead of median (IQR) due to the small sample size in the unclassified subtype*.

*GBS, Guillain–Barré syndrome; AIDP, acute inflammatory demyelinating polyneuropathy; AMAN, acute motor axonal neuropathy; AMSAN, acute motor and sensory axonal neuropathy; MFS, Miller Fisher syndrome; NIND, non-inflammatory neurological disorders; HC, healthy control; CSF, cerebrospinal fluid; UA, uric acid; SD, standard deviation; IQR, interquartile range*.

### Data and Sample Collection

The demographic features, preceding infections, history of disease, GBS disability scores, antiganglioside (GM1/GQ1b) antibodies status, electrophysiology, and CSF parameters of enrolled GBS patients were obtained from our GBS database. The preceding infections of the GBS patients were determined based on the influenza-like syndrome (such as fever and respiratory signs) and gastrointestinal tract infection (usually diarrhea) within previous 4 weeks before the onset of weakness. The severity of disease was evaluated using the GBS disability score (31) at nadir by at least two experienced neurologists independently. CSF samples from the 43 patients with GBS were collected during the acute phase, and CSF samples from 25 patients with NIND were also collected as controls. All CSF samples were immediately aliquoted and stored at −80°C after lumbar puncture until further analysis. Serum samples from 43 patients with GBS, 25 patients with NIND, and 30 healthy controls were collected and stored at −80°C until analysis. All the samples from patients and controls were obtained undisturbedly after an overnight fasting in order to minimize the effects on uric acid.

### Measurement of UA Levels

Serum and CSF UA levels were measured using uricase-based methods with a LABOSPECT 008 α Hitachi Automatic Analyzer (Hitachi High-Technologies, Tokyo, Japan) according to the manufacturer's instructions. The normal range of serum UA levels in our hospital is 140–414 μmol/L.

### Statistical Analysis

All analyses were performed using SPSS, version20 (IBM Corp., Armonk, NY, USA) and GraphPad Prism, version 7 (GraphPad Software Inc., San Diego, CA, USA). The quantitative data were presented as mean ± standard deviation (SD) or median with interquartile range (IQR) depending on whether the data were normally distributed. In summary, age was normally distributed. The UA levels in CSF and serum, GBS disability scores, and CSF parameters were not normally distributed. A *p* < 0.05 was considered statistically significant. Mann–Whitney *U*-tests were conducted for quantitative data analysis between two groups and Kruskal–Wallis *H*-tests for three or more groups. Correlation between CSF UA levels and age, serum UA levels, CSF parameters, and GBS disability scores were evaluated by Spearman's rank correlation analysis. The one-way ANOVA was used to compare age or other normally distributed continuous data among three or more groups. The chi-square tests or Fisher exact tests were conducted to compare qualitative variables. The Bonferroni correction was performed to adjust *p*-values for multiple testing.

## Results

### Demographic and Clinical Features of Subjects

Demographic and clinical features of enrolled cases in the present study are summarized in Table 1. There were no differences in age or gender among patients with GBS, NIND, and healthy controls (*p* = 0.284 for age and *p* = 0.906 for gender). In patients with GBS, 55.8% had preceding infections, 44.2% had cranial nerve involvement, 65.1% had antiganglioside antibodies positive, and 90.7% had albuminocytologic dissociation.

### Increased CSF UA Levels in GBS and AIDP Compared With NIND

**Figure 1:**
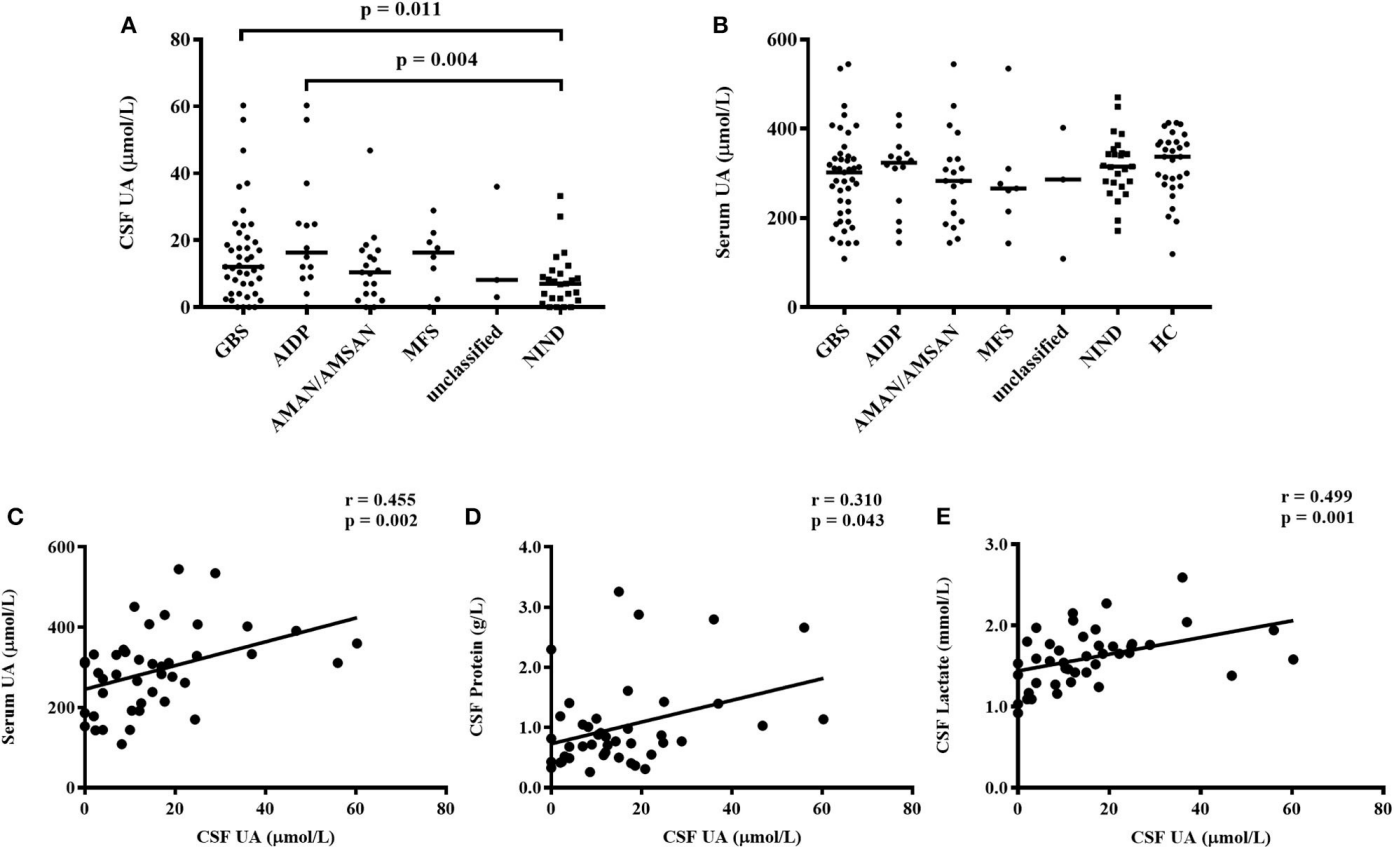
CSF and serum UA levels in subtypes of GBS, NIND, and HC. **(A)** CSF UA levels were increased significantly in patients with GBS (*p* = 0.011) and AIDP subtype (*p* = 0.004) compared with NIND. **(B)** The difference of serum UA levels among patients with GBS, NIND, and HC were not statistically significant (*p* = 0.175). **(C)** Correlation between levels of CSF UA and serum UA, adjusted *p* = 0.022 after the Bonferroni correction. **(D)** Correlation between CSF UA and CSF protein, adjusted *p* = 0.473 after the Bonferroni correction. **(E)** Correlation between CSF UA and CSF lactate, adjusted *p* = 0.011 after the Bonferroni correction. GBS, Guillain–Barré syndrome; AIDP, acute inflammatory demyelinating polyneuropathy; AMAN, acute motor axonal neuropathy; AMSAN, acute motor and sensory axonal neuropathy; MFS, Miller Fisher syndrome; NIND, non-inflammatory neurological disorders; HC, healthy control; CSF, cerebrospinal fluid; UA, uric acid.

In the present study, CSF UA levels were significantly higher in patients with GBS compared with NIND [12.10 (4.00, 20.80) μmol/L vs. 7.00 (2.30, 10.50) μmol/L, *p* = 0.011, Figure 1A]. In subtypes of GBS, patients with AIDP, but not other subtypes, had increased CSF UA levels compared with NIND [16.35 (8.90, 28.00) μmol/L vs. 7.00 (2.30, 10.50) μmol/L, *p* = 0.004, Figure 1A]. However, no differences in CSF UA levels were found among the four subtypes of GBS [16.35 (8.90, 28.00) μmol/L for AIDP, 10.40 (4.00, 17.00) μmol/L for AMAN/AMSAN, 17.70 (2.40, 22.20) μmol/L for MFS, 8.20 (3.00, 36.00) μmol/L for unclassified, *p* = 0.285].

### No Significant Differences in Serum UA Levels Among GBS, NIND, and Healthy Controls

The serum UA levels in GBS, NINID, and healthy controls were 302.00 (210.60, 338.00) μmol/L, 315.00 (274.50, 349.50) μmol/L, and 337.00 (274.00, 370.00) μmol/L, respectively. However, there were no significant differences in serum UA levels among these three groups (*p* = 0.175, Figure 1B), neither among four subtypes of GBS (*p* = 0.650, Figure 1B). No significant differences were found in serum UA levels when comparing each subtype of GBS with NIND or healthy controls separately.

### UA Levels and Different Clinical Characteristics of Patients With GBS

To investigate the UA levels in GBS patients with different clinical characteristics, 43 patients with GBS were further divided into two subgroups by gender, preceding infections, antiganglioside antibodies, GBS disability scores, cranial nerve involvement, and nerve conduction study (NCS) results (excluded two patients whose NCS results were not available). As shown in Table 2, the CSF UA levels in patients with demyelination were significantly higher than in patients without demyelination (*p* = 0.022). The serum UA levels of female GBS patients were significantly lower than male patients (*p* = 0.011). However, after multiple testing corrections (adjusted *p*-value = *p*-value^*^7 characteristics), neither of these differences remained significant. The differences in UA levels were not statistically significant in other clinical characteristics in patients with GBS.

**Table 2:** CSF and serum UA levels in GBS patients with different clinical characteristics.

**Clinical characteristics**	**CSF UA (μmol/L), median (IQR)**	***P*-value**	**Adjusted *p*-value**	**Serum UA (μmol/L), median (IQR)**	***P*-value**	**Adjusted *p*-value**
**Gender**						
Female (*n* = 22)	12.30 (4.00, 20.65)	0.961	1.000	225.30 (176.00, 317.90)	0.011^*^	0.077
Male (*n* = 21)	12.00 (5.00, 21.50)			319.00 (284.00, 352.05)		
**Preceding infections**						
With (*n* = 24)	14.65 (4.75, 20.45)	1.000	1.000	309.35 (203.30, 336.75)	0.732	1.000
Without (*n* = 19)	11.60 (4.00, 24.80)			286.00 (210.60, 344.40)		
**Antiganglioside antibodies**						
Positive (*n* = 28)	11.80 (4.75, 20.45)	0.683	1.000	294.00 (223.33, 336.75)	0.665	1.000
Negative (*n* = 15)	15.00 (4.00, 24.40)			308.70 (170.00, 402.00)		
**GBS disability scores**						
<3 (*n* = 24)	15.00 (5.05, 21.85)	0.633	1.000	292.50 (202.85, 399.25)	0.826	1.000
≥3 (*n* = 19)	10.40 (4.00, 18.60)			311.00 (210.60, 331.00)		
**Cranial nerve involvement**						
With (*n* = 19)	15.00 (2.40, 22.20)	0.797	1.000	308.70 (238.90, 332.00)	0.883	1.000
Without (*n* = 24)	12.05 (7.00, 20.03)			292.50 (191.95, 383.18)		
**Nerve conduction study**						
**Axonal injury**						
With (*n* = 34)	13.40 (6.25, 24.50)	0.290	1.000	309.35 (206.05, 348.23)	0.703	1.000
Without (*n* = 7)	11.60 (2.40, 17.70)			286.00 (261.50, 319.00)		
**Demyelination**						
With (*n* = 26)	15.00 (9.75, 24.50)	0.022^*^	0.154	312.60 (273.90, 370.28)	0.066	0.462
Without (*n* = 15)	4.00 (2.00, 17.00)			261.50 (186.00, 331.00)		
**F-wave abnormalities**						
With (*n* = 38)	12.05 (4.00, 21.70)			309.35 (229.65, 348.23)		
Without (n = 3)	18.60 (2.40, 22.20)^#^			261.50 (143.00, 311.20)^#^		

The Bonferroni correction was performed to adjust p values for multiple testing correction (adjusted p-value = p-value*7 characteristics).

* *p < 0.05 before multiple testing correction*.

# *Data was presented as median (range) instead of median (IQR) and no statistical analysis was performed due to the small sample size*.

*Guillain–Barré syndrome; CSF, cerebrospinal fluid; UA, uric acid; IQR, interquartile range*.

### Correlations Between CSF UA Levels and Age, Serum UA Levels, GBS Disability Scores, and CSF Parameters in Patients With GBS

In the present study, Spearman's rank correlation analysis was conducted to investigate the relationship between CSF UA levels and age, serum UA levels, GBS disability scores, and CSF parameters in patients with GBS. CSF UA levels were positively correlated with serum UA levels (*r* = 0.455, *p* = 0.002; Figure 1C), CSF protein (*r* = 0.310, *p* = 0.043; Figure 1D), and CSF lactate (*r* = 0.499, *p* = 0.001; Figure 1E). After using the Bonferroni correction for multiple testing (adjusted *p*-value = *p*-value^*^11 characteristics), the correlations with serum UA levels (adjusted *p* = 0.022) and CSF lactate (adjusted *p* = 0.011) were still significant. However, no significant correlations were found between CSF UA levels and age (*r* = 0.210, *p* = 0.176), GBS disability scores (*r* = −0.013, *p* = 0.934), CSF white blood cells (*r* = 0.292, *p* = 0.057), CSF lactate dehydrogenase (*r* = 0.275, *p* = 0.074), CSF adenosine deaminase (*r* = 0.259, *p* = 0.094), CSF high-sensitive C-reactive protein (*r* = 0.060, *p* = 0.705), CSF glucose (*r* = 0.133, *p* = 0.394), or CSF chloride (*r* = −0.147, *p* = 0.348).

## Discussion

There has been a growing interest in the role of UA in the nervous system due to the potential for antioxidant therapy and reflection of oxidative stress and purine catabolism in neurological diseases (15, 17, 32, 33). The results of this study showed that the UA levels in the CSF were significantly elevated in patients with GBS, especially in AIDP, when compared with NIND, but no differences in serum UA levels among GBS, NIND, and healthy controls. These findings were consistent with those of Becker et al. who found markedly increased CSF urate in GBS and bacterial meningitis (27). Unlike Su et al. (26), we did not observe the reduced serum UA levels in patients with GBS. We speculated that the increasing CSF UA levels in patients with GBS may be related to oxidative stress and metabolism of purine and ATP.

UA is considered a natural antioxidant and believed to have neuroprotective functions by counteracting the peroxynitrite-mediated oxidative stress (4, 32). Oxidative stress and impaired antioxidant defense have been established to be involved in the pathogenesis of neuroinflammatory diseases (21–24). Peroxynitrite, a highly reactive oxidant molecule, is notorious for causing cell damage in various conditions including inflammation and neurodegeneration (34). Reactive oxygen species and peroxynitrite may result in cell and tissue damage directly by the oxidation of DNA, proteins, and lipids (35). Activation of glia and macrophages in neuroinflammation may induce oxidative stress by generation of more oxygen and nitrogen free radicals (36). The function of the mitochondrial respiratory chain may be affected by peroxynitrite and other reactive species, which may contribute to the spread of oxidative damage and impaired ATP metabolism (35). As the end metabolite of purine catabolism, UA is related to nucleic acid catabolism and assumed to be associated with adenosine and ATP metabolism, which may be a potential biomarker of energy condition (15, 37).

The CSF UA levels in CNS neuroinflammatory diseases were inconsistent in different studies. Some studies found no significant difference in CSF UA levels between MS patients and controls (27, 38, 39), while one study found lower CSF UA levels in patients with MS (40). Elevated CSF UA levels were observed in patients with MS and NMOSD, which may reflect oxidative stress and excitotoxicity (16, 17). In experimental autoimmune encephalomyelitis, an animal model of MS, UA plays a therapeutic role through inhibiting peroxynitrite-mediated toxicity, maintaining the integrity of the blood–brain barrier, and preventing immune cell invasion (37, 41, 42). Therefore, in view of compensatory mechanisms, UA might be recruited as the antioxidant in CSF under disease condition (27). In the study of Amorini et al. (15), the elevated levels of UA, purine metabolites, and creatinine in both CSF and serum in patients with MS were interpreted as enhanced purine catabolism and energy imbalance, which questioned the view that UA mainly acted as an antioxidant in neurological diseases. However, taking into account the damage of mitochondria and the impaired ATP metabolism in oxidative stress (35), reflecting purine and energy metabolism is not contradictory with UA as an antioxidant. The inconsistent results of different studies might be related to the difference in controls, sample sizes, and measurement methods.

Little attention has been paid to UA in peripheral nerves system diseases such as GBS. In this study, the elevated CSF UA levels in patients with GBS may be interpreted as the compensatory reaction of antioxidant activity against the increasing oxidative stress. In the study of Tang et al. (23), lipophilic antioxidants in GBS patients were decreased, which indicating a reduced resistance to oxidative damage. Kumar et al. (21) found that free radical toxicity in GBS could lead to a compensatory increase in antioxidants such as plasma vitamin E and erythrocyte glutathione. In addition, in experimental autoimmune neuritis, the animal model of GBS, enhanced glycolysis has been proven (43). As a product of glycolysis, lactate in CSF is believed to represent energy metabolism (44). In the present study, UA levels in CSF were positively correlated with CSF lactate, indicating an association between the energy or ATP metabolism and UA levels in patients with GBS. Thus, it is plausible to presume that increased CSF UA levels may also reflect purine metabolism and energy requirements in GBS. Furthermore, the impaired blood–CSF or blood–nerve barrier may also be part of the reason for the increase in CSF UA levels (10, 27). Albuminocytologic dissociation, a characteristic manifestation in CSF of patients with GBS, has been supposed to be caused by the disruption of the blood–nerve barrier on account of inflammation at the nerve root (45). Reactive oxygen species were reported to account for changes in tight junction of endothelium and loss of integrity in the blood–CSF barrier (36). In this study, the UA levels in CSF were positively correlated with serum UA, indicating that CSF UA may be partially determined by serum UA and the impaired blood–CSF or blood–nerve barrier in GBS, which is consistent with previous studies in MS and NMOSD (16, 38). Female GBS patients had lower serum UA levels in the present study, but there was no gender difference in CSF, which is in line with the study of Shu et al. in NMOSD (16). Lower serum UA levels in female have been identified in MS, NMOSD, and healthy controls (25, 46). The reason for the gender differences in UA levels was not clear (46). It was speculated that the lower levels of UA in female were due to the increase in fractional excretion of UA induced by estrogen (47).

Notably, unlike other subtypes of GBS, patients with AIDP showed significant increase in CSF UA levels compared with NIND. Within GBS patients, cases whose NCS results indicted demyelination had higher levels of CSF UA than cases without demyelination, although the difference was not significant after multiple testing correction. The pathophysiological differences in subtypes of GBS have been extensively studied (19). Antibodies against gangliosides (mainly GM1 and GD1a) in exposed nerve membranes at nodes of Ranvier and nerve terminals are specific biomarkers for AMAN and axonal loss type. Antibodies against GQ1b ganglioside are most common in MFS. However, antibody biomarkers have not been identified in AIDP. It is believed that nerve-specific T cell and a wider range of antinerve autoantibodies, as well as complement activation and macrophage scavenging may play an important role in demyelination of AIDP (19). The myelin sheath, largely composed of lipids, is vulnerable to oxidative stress (23, 24). The multiple layers of lipid membranes in myelin sheath could be easily impaired by lipid peroxidation (36). It has been proven that demyelination can be aggravated by oxidative stress and lipid peroxidation (21). Activated macrophages can produce more oxygen and nitrogen free radicals. The inflammatory environment of demyelinating might result in an increase in reactive oxygen and nitrogen species (36). In turn, lipid peroxidation of myelin may contribute to immune pathogenesis of GBS by generation of lipid-derived inflammatory mediators and proinflammatory cytokines (21, 36). The vulnerability to lipid peroxidation in lipid-rich myelin sheath and the increased compensatory antioxidant activity may be the reason for elevated CSF UA levels in AIDP. High levels of CSF UA were also observed in patients of MFS, although this was not statistically significant. MFS is mainly implicated in axolemma rather than the myelin sheath, which is associated with anti-GQ1b antibodies (48). Antibody-mediated inflammation may also lead to oxidative stress and lipid peroxidation of axolemma in MFS. The antioxidant activity against lipid peroxidation in the reticular formation and cerebellum, which possibly expressed GQ1b, may account for the high CSF UA levels in MFS (36, 48).

CSF UA levels are affected by multiple factors (27), and the role of UA in GBS remains uncertain. This study has revealed increased CSF UA levels in patients with GBS and AIDP and showed a prospect that elevated CSF UA may be associated with demyelination. However, no correlations with disease severity limited the CSF UA as a biomarker in GBS. Based on the above previous research findings and assumptions and the results of the present study, we speculated that increased UA concentration in CSF may reflect oxidative stress and enhanced purine or ATP metabolism in patients with GBS.

Several limitations to this study need to be acknowledged. First, the sample size is small in the present study, especially when considering the subtypes of GBS, which may be the limit for firm conclusions. Future research requires a larger sample size and a sufficient number of cases in subtypes of GBS. Second, allantoin, a product of the oxidation of UA, and other products of purine, were not detected in this study. Future work should therefore include allantoin and purine metabolites such as hypoxanthine and xanthine to evaluate oxidative stress and purine catabolism in patients with GBS. Third, although the relationship between CSF UA and demyelination has been proposed with evidence in this study, further studies are needed to verify the lipid peroxidation in GBS probably through estimating malondialdehyde, a biomarker of lipid peroxidation and oxidative stress (49). Fourth, the GBS patients included in this study were all in the acute phase; future research should focus on CSF UA levels in patients with different disease stages.

## Conclusion

In summary, this study has shown that the levels of UA in CSF were significantly increased in patients with GBS and AIDP, suggesting that CSF UA may be related to the pathogenesis of demyelination in patients with GBS and may be partially determined by serum UA and the impaired blood–nerve barrier. As a natural antioxidant and the end metabolite of purine catabolism, elevated UA levels in CSF may reflect oxidative stress and enhance purine or ATP metabolism in GBS.

## Data Availability Statement

The raw data supporting the conclusions of this article will be made available by the authors, without undue reservation.

## Ethics Statement

The studies involving human participants were reviewed and approved by the Medical Research Ethics Committee at Tianjin Medical University General Hospital. The patients/participants provided their written informed consent to participate in this study.

## Author Contributions

LY, X-BT, and S-HC contributed to the design and data interpretation. S-HC and X-BT contributed to the drafting of the manuscript. S-HC, JW, and L-JZ contributed to data analysis. M-QL, YQ, C-NH, and C-LG contributed to data collection, case diagnosis, and confirmation. L-SS contributed to sample analysis. LY, X-BT, and D-QZ contributed to critical review. All authors contributed to the article and approved the submitted version.

## Conflict of Interest

The authors declare that the research was conducted in the absence of any commercial or financial relationships that could be construed as a potential conflict of interest.
